# Trade‐off between reproductive and anti‐competitor abilities in an insect–parasitic nematode–bacteria symbiosis

**DOI:** 10.1002/ece3.4538

**Published:** 2018-10-18

**Authors:** Sofia Bertoloni Meli, Farrah Bashey

**Affiliations:** ^1^ Department of Biology Indiana University Bloomington Indiana

**Keywords:** allelopathy, competition, context‐dependent fitness, diversity, mutualism

## Abstract

Mutualistic symbionts can provide diverse benefits to their hosts and often supply key trait variation for host adaptation. The bacterial symbionts of entomopathogenic nematodes play a crucial role in successful colonization of and reproduction in the insect host. Additionally, these symbionts can produce a diverse array of antimicrobial compounds to deter within‐host competitors. Natural isolates of the symbiont, *Xenorhabdus bovienii,* show considerable variation in their ability to target sympatric competitors via bacteriocins, which can inhibit the growth of sensitive *Xenorhabdus* strains. Both the bacteria and its nematode partner have been shown to benefit from bacteriocin production when within‐host competition with a sensitive competitor occurs. Despite this benefit, several isolates of *Xenorhabdus* do not inhibit sympatric strains. To understand how this variation in allelopathy could be maintained, we tested the hypothesis that inhibiting isolates face a reproductive cost in the absence of competition. We tested this hypothesis by examining the reproductive success of inhibiting and non‐inhibiting isolates coupled with their natural nematode host in a non‐competitive context. We found that nematodes carrying non‐inhibitors killed the insect host more rapidly and were more likely to successfully reproduce than nematodes carrying inhibitors. Lower reproductive success of inhibiting isolates was repeatable across nematode generations and across insect host species. However, no difference in insect mortality was observed between inhibiting and non‐inhibiting isolates when bacteria were injected into insects without their nematode partners. Our results indicate a trade‐off between the competitive and reproductive roles of symbionts, such that inhibiting isolates, which are better in the face of within‐host competition, pay a reproductive cost in the absence of competition. Furthermore, our results support the hypothesis that symbiont variation within populations can be maintained through context‐dependent fitness benefits conferred to their hosts. As such, our study offers novel insights into the selective forces maintaining variation within a single host–symbiont population and highlights the role of competition in mutualism evolution.

## 1 INTRODUCTION

Identifying the adaptive context underlying trait variation is a central aim in evolutionary ecology. Often, the maintenance of phenotypic and genetic trait variation is linked to context‐specific costs and benefits. Both predator‐induced changes in animal morphology (Laforsch & Tollrian, 2004; Lively, 1986) and herbivore defenses in plants (Strauss, Rudgers, Lau, & Irwin, 2002) have been examined under the framework of context‐specific benefits in the presence of predators and costs in their absence. Like predation, competition favors specific phenotypes that can be costly in the absence of competition. For example, increased production of sinigrin, an allelopathic compound produced by *Brassica nigra*, provides an advantage in competition with other plant species, but is costly to growth in the absence of interspecific competition (Lankau, 2008). Such tensions between fitness effects in different competitive environments can maintain genetic variation (Lankau & Strauss, 2007).

Furthermore, organismal traits are often the product of interactions between the organism and its microbial symbionts. Symbionts can increase the abiotic tolerances of their hosts, provide access to vital nutrients, aid in successful reproduction, and protect their eukaryotic host from predators and parasites (Brock, Read, Bozhchenko, Queller, & Strassmann, 2013; Clay & Fox, 2014; Feldhaar, 2011). Thus, variation in the selective environment can also affect the symbionts within a eukaryotic host. For example, the aphid symbiont *Hamiltonella defensa*, which confers resistance to a wasp parasite, increases in frequency when the parasite is common. However, when parasitism is low, the frequency of *H. defensa* drops in aphid populations, indicating a cost of retaining this symbiont (Oliver, Campos, Moran, & Hunter, 2008). Even within a single population at a specific time, different symbiont genotypes have been found to differentially affect host fitness (Heath, 2010). Explaining this variation remains an outstanding gap in our understanding of how mutualisms evolve (Heath & Stinchcombe, 2014).

**Figure 1:**
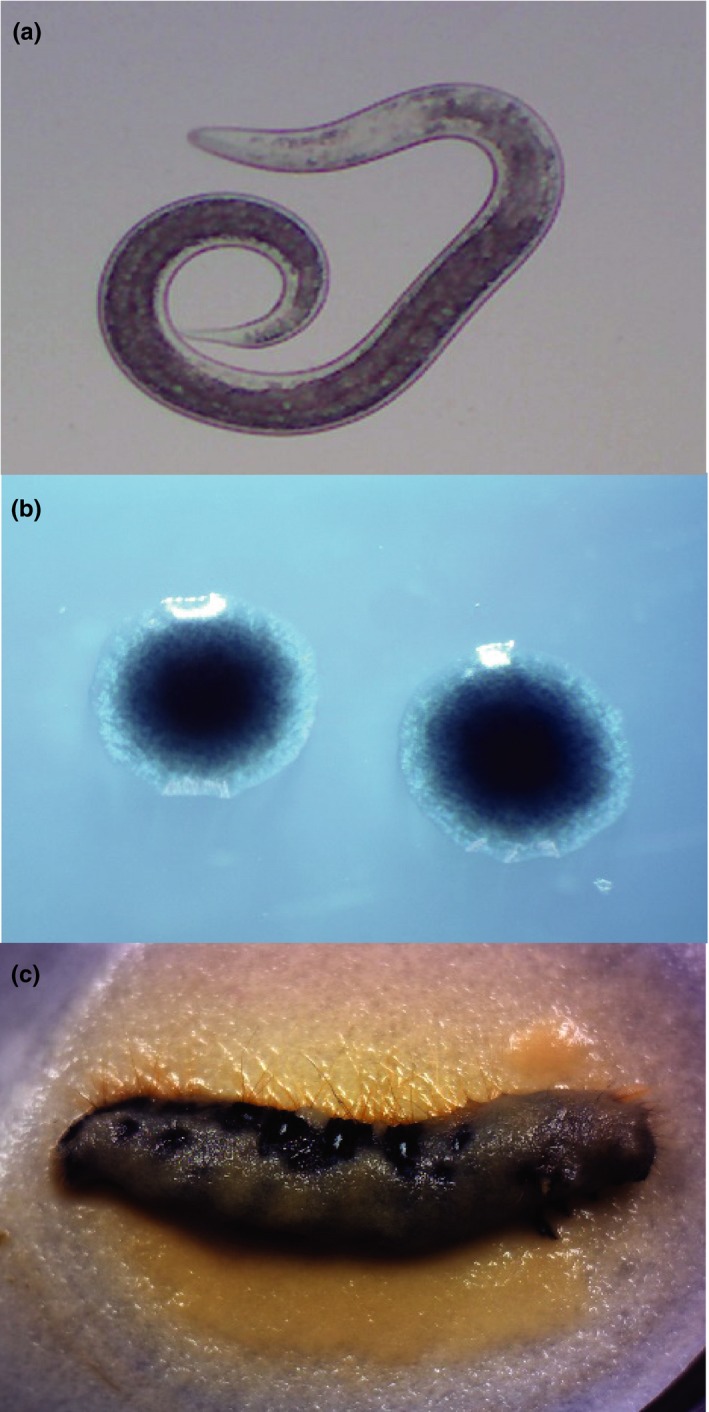
The infective juvenile of the entomopathogenic nematode, *Steinernema affine* (a), persists free‐living and non‐feeding in the soil and carries its symbiotic bacterial partner, *Xenorhabdus bovienii* (b), shown here as colonies on an agar plate. The mutualistic partners infect, kill, and reproduce inside insect hosts, such as the Eastern tent caterpillar *Malacosoma americanum* (c), shown here with thousands of newly emerging infective juvenile nematodes

Here, we examine the potential for variation in a microbial symbiont, *Xenorhabdus bovienii,* to be maintained by the context‐specific fitness benefits conferred to its mutualistic partner, the nematode *Steinernema affine* (Figure 1). In nature, there is an obligate association between these two species. The bacteria depend on the nematode for vectoring among insect hosts, while the nematodes depend on their symbiont for effective colonization of and reproduction in an insect host (Poinar & Thomas, 1966; Sicard et al., 2003). Additionally, *Xenorhabdus* bacteria produce a suite of secondary metabolites and defensive compounds that protect the insect cadaver from fungal and bacterial saprophytes (Forst & Nealson, 1996). One defensive compound produced by *Xenorhabdus* is a highly specific phage tail‐like anti‐competitor toxin known as a bacteriocin (Boemare, Boyer‐Giglio, Thaler, Akhurst, & Brehelin, 1992). This molecule inhibits the growth of other closely related bacteria (i.e. those in the genera *Xenorhabdus*,* Photorhabdus,* and *Proteus*) by binding to membrane‐bound receptors and depolarizing the membrane (Morales‐Soto & Forst, 2011). Within the insect host, bacteriocin‐based inhibition provides a competitive advantage to the producing lineage when coinfecting with a sensitive strain (Bashey, Young, Hawlena, & Lively, 2012). Furthermore, when nematodes compete within the insect, those carrying symbionts that can inhibit the symbionts of their competitors are more likely to successfully reproduce and transmit propagules (Bashey, Hawlena, & Lively, 2013).

Despite the advantage conferred by bacteriocin production, several non‐inhibiting clones of *X. bovienii* have been isolated from nature (Hawlena, Bashey, & Lively, 2012). Is this variation due to context‐specific fitness benefits of this trait? The bacteriocins produced by *Xenorhabdus* are large molecules, which are thought to be released by lysis (Morales‐Soto, Gaudriault, Ogier, Thappeta, & Forst, 2012). Moreover, when competition occurs between two isolates which are not sensitive to each other's bacteriocin, the isolate that is able to establish an infection more quickly is competitively dominant (Bashey et al., 2013). Thus, there may be a trade‐off between the benefit of bacteriocin production in the context of competition with a sensitive competitor and the costs of bacteriocin production in the absence of competition. Here, we test the hypothesis that bacteriocin production is costly in the absence of competition by examining several fitness components of nematodes carrying either inhibiting or non‐inhibiting bacteria in single‐strain infections. We predict that inhibiting strains will have lower fitness than non‐inhibiting strains in the absence of competition. We also examined each type of bacteria's infection success in the absence of their nematode partners to see whether the bacterial symbiont alone affects the rate of host killing, a key fitness component for these obligately killing parasites. Finally, we tested whether these effects were repeatable with nematode age, passage in the laboratory, and across different host species to gain insights into the mechanisms behind these differential symbiont effects.

## 2 MATERIALS AND METHODS

### 2.1 Nematode ‐bacteria mutualism

*Steinernema* nematodes are free living in the soil as juveniles and carry their symbiotic *Xenorhabdus* bacteria in a receptacle within the intestine (Snyder, Stock, Kim, Flores‐Lara, & Forst, 2007). Non‐feeding in this transmission stage, the nematodes must infect an insect host to develop to adulthood. Once inside the insect, the bacteria are released from the nematode. The nematodes and bacteria both contribute to the killing of the insect host (Brivio, Pagani, & Restelli, 2002; Goetz, Boman, & Boman, 1981; Simões, 2000). After host death, the nematodes reproduce within the cadaver, their fitness greatly facilitated by the presence of their bacteria (Sicard et al., 2003). As the host resources become exhausted, the bacteria reassociate with the nematode and they emerge from the insect (Martens, Heungens, & Goodrich‐Blair, 2003; Popiel, Grove, & Friedman, 1989; Wang & Bedding, 1996). Both partners benefit from the association, as *Xenorhabdus* bacteria have limited ability to survive in the soil without their nematode (Morgan, Kuntzelmann, Tavernor, Ousley, & Winstanley, 1997).

### 2.2 Isolates used in this study

Nematodes and bacteria were isolated from nine soil samples collected from Indiana University Research and Teaching Preserve, Moore's Creek, Monroe County, Indiana, in October 2011. Soil samples (8 cm in diameter by 5 cm in depth) were along a 300‐meter transect from the sites described by Hawlena, Bashey, and Lively (2010). Each soil sample was exposed to *Galleria mellonella* caterpillars, and the emerging nematodes were identified as *Steinernema affine* by sequencing 28S rRNA gene (Stock, Campbell, & Nadler, 2001). Each of the nine nematode isolates used in this study originated from a single caterpillar exposed to a unique one of the nine soil samples.

Bacteria were further isolated from each nematode isolate following the methods described by Hawlena, Bashey, Mendes Soares, and Lively (2010) and were identified as *Xenorhabdus bovienii* using 16S rRNA gene (Tailliez, Pages, Ginibre, & Boemare, 2006). Multiple bacterial isolates were saved for each nematode isolate and stored as freezer stocks in 20% glycerol at −80°C. Bacterial isolates from each of the nine soil samples were tested for their ability to inhibit sympatric *Xenorhabdus* bacteria using inhibition assays as described in Hawlena, Bashey, Mendes Soares, et al. (2010). Briefly, 0.5 µg/ml mitomycin C (Sigma‐Aldrich) was added to exponential phase cultures to induce bacteriocin production and incubated overnight at 28°C. Cell‐free supernatant was obtained from each culture by centrifugation (15 min at 4,500 g) followed by filtering via 0.45‐µm HT Tuffryn membrane and stored at 4°C. The bacteriocin activity of the supernatant was tested by spotting 10 µl onto a nutrient soft agar (0.5% agar) sowed with 2% (v/v) of stationary‐phase liquid culture of a recipient colony. Each bacterial isolate was induced to produce bacteriocin on two separate dates, and the resulting bacteriocin was tested against sympatric *Xenorhabdus spp.* isolates associated with four different nematode species collected from 28 soil samples along the 300‐m transect over two sampling years. Each isolate was also tested for its ability to resist inhibition by the same set of isolates. These isolates represent the diversity in this genus that we have characterized at this site. Prior work has established that little variation exists within a soil sample in bacteriocin phenotype, although samples collected just a few meters apart have distinct phenotypes (Hawlena, Bashey, & Lively, 2010; Hawlena, Bashey, Mendes Soares, et al., 2010). Self‐tests whereby the supernatant derived from an isolate was tested for growth inhibition against itself showed no inhibition as expected due to self‐immunity typical of bacteriocin‐mediated killing (Riley & Chavan, 2007). Based on these assays, the nine *S. affine* nematode isolates obtained from the 2011 sampling were characterized as carrying inhibiting or non‐inhibiting symbionts (Table 1) and used to examine the trade‐off between inhibition status and reproductive fitness in the absence of competition.

**Table 1:** Symbiont competitive ability assessed by growth inhibition of sympatric *Xenorhabdus* isolates

Isolate name[Link]	*X. bovienii* [Link]				*X. koppenhoeferi* [Link]
	*S. affine* (12)[Link]	Unidentified (2)	*S. kraussei* (6)	*S. texanum* (3)	*S. costaricense* (6)
Non‐inhibiting bacterial symbionts					
202	0[Link]	0	0	0	0
214	0	0	0	0	0
226	0	0	0	0	0
235	0	0	0	0	0
Inhibiting bacterial symbionts					
211	1	1	2	0	0
216	1	6	3	3	4
218	2	5	3	3	4
228	1	9	3	0	2
255	7	1	4	3	4

Each *S. affine* nematode isolate was extracted from a separate soil sample. Two *X. bovienii* colonies were isolated from each nematode isolate/soil sample and tested for bacteriocin activity against similarly collected sympatric *Xenorhabdus* isolates.

In addition to *S. affine*,* X. bovienii* bacteria are associated with *S. kraussei* and *S. texanum* nematodes in this community. *X. bovienii* was also isolated from in two cases where too few nematodes were obtained for species identification.

Another sympatric nematode competitor of *S. affine* is *S. costaricense* which is associated with *X. koppenhoeferi* bacteria.

Numbers in ( ) indicate the number of soil samples from which bacteria were tested for sensitivity.

Numbers in each cell indicate the number of soil samples where inhibitions were detected

### 2.3 Effect of symbiont status on nematode parasitic success

In order to test whether there was a reproductive cost for the nematode associated with the inhibiting phenotype of its symbiont, each nematode isolate was assayed for speed in host killing and its ability to reproduce. In order to successfully reproduce, the nematode isolate must kill the insect host. Nematodes that establish an infection more rapidly can gain access to nutrients and outcompete other slower‐killing nematodes, therefore speed of host killing is an important component of reproductive success (Bashey, Reynolds, Sarin, & Young, 2011). We predicted that non‐inhibiting isolates would kill insect hosts faster than the inhibiting isolates and have a higher probability of host death than the inhibitors. Moreover, we predicted that non‐inhibiting isolates would be more effective at reproducing, and therefore, we predicted they would have a higher probability of emergence and produce more or better quality offspring than inhibiting isolates.

Prior to the assay, each nematode isolate was passaged in the laboratory through *G. mellonella* hosts (Vanderhorst Wholesale) to remove environmental effects of dose or nematode condition (e.g., age and energy reserves) associated with the initial soil sampling. For passaging, each isolate was used to infect 20 *G. mellonella* as described for the assays below and the nematodes emerging from five caterpillars were combined to form the infective dose for the assay. For the assay, each isolate was used to infect 20 caterpillars at a dose of 100 nematodes per caterpillar for a total of 180 caterpillars per assay. Caterpillars were placed in a 60 × 20 mm petri dish lined with filter paper (Whatman no. 1) and moistened with 0.5 ml of dH_2_O. Each caterpillar was infected by placing water containing the infective dose of nematodes on its dorsum and kept at 20°C. During mortality checks, caterpillars were touched with a probe to be assessed for movement. If they did not move, they were recorded as dead. Caterpillar death usually occurred within the first 150 hr post infection. Approximately 7 days post infection, dead caterpillars were transferred to white traps (Bashey, Morran, & Lively, 2007; White, 1927) for collection of nematodes. Nematode emergence was monitored using a dissecting microscope. Nematodes were stored at 8°C in dH_2_O.

Nematode samples from each caterpillar were counted using a dissecting microscope, and the total number of nematodes was estimated based on total volumetric subsampling. Size of the infective juveniles may be an important factor influencing reproductive success as infective juveniles persist in the soil in a non‐feeding state before infecting a new host; thus, larger nematodes with more energy stores are more likely to survive (Qui & Bedding, 2000). Furthermore, there can be a trade‐off between the size and number of nematodes that can emerge from a given size host (Therese & Bashey, 2012). From each of the nine isolates, images of five nematodes from each caterpillar were captured at 100×, and ImageJ was used to determine the area of each nematode.

### 2.4 Bacterial mortality assay

To determine whether differences found across nematode stocks carrying inhibiting versus non‐inhibiting bacteria could be attributed to the bacteria alone, we examined the infection success and mortality rate of insects infected with bacteria from each nematode isolate. We predicted that if there was a cost associated with bacteriocin‐based inhibition, then inhibiting isolates would be slower at killing the insect host.

Each of the nine isolates was used separately to infect insect hosts. A 30‐gauge needle was used to inject each caterpillar with 20 µl containing stationary‐phase bacteria. Dose was set to mimic the dose in the nematode infections (approximately 100 nematodes with 100 bacterial cells in each). Caterpillars were monitored for death 10 times over 46 hr. Three replicate mortality assays were performed. In each assay, each isolate was used to infect 20 caterpillars, resulting in a total of 540 caterpillars for the three assays.

### 2.5 Repeatability of isolate differences in nematode success

As our characterization of each nematode isolate was based on a single trait, i.e. the bacteriocin phenotype of its bacterial symbiont, we wanted to examine the robustness of the observed reproductive differences across isolates to variation in condition of the nematodes and their caterpillar hosts. Thus, we repeated our assessment of nematode parasitic ability using nematodes of different ages and from different host passages (Table 2) in order to determine the consistency of the differences between inhibiting and non‐inhibiting isolates. Nematodes from the same passage at different times (e.g. Assay 1 vs. 2) and from subsequent passages (e.g. Assay 2 vs. 4) were examined. Additionally, infection patterns in a different host insect species, *Malacosoma americanum* (eastern tent caterpillar), were examined in Assay 4. Based on the results from Assay 1, we focused on the overall probability of emergence, which is the probability that nematodes emerged from an insect experimentally exposed to nematodes. In each assay, 20 caterpillars were exposed to nematodes of each isolate for a total of 180 caterpillars per assay and 1,080 caterpillars total.

**Table 2:** Characterization of repeatability assays

Assay	Nematode generation	Host species	Age (in months) of nematodes at time of infection	Nematode source
1	F2	*Galleria*	2	Isolates passaged twice through hosts in the lab
2	F2	*Galleria*	11	Same as Assay 1
				
3	F3	*Galleria*	1	Nematodes emerging from Assay 2
4a	F3	*Galleria*	9	Nematodes emerging from Assay 2
4b	F3	*Malacosoma*	9	Nematodes emerging from Assay 2

### 2.6 Statistical analysis

All analyses were conducted using SAS v. 9.4. T‐tests were used to examine the difference in resistance between inhibiting and non‐inhibiting bacterial isolates. To examine the difference in time of host death in both the nematode infections and the bacterial mortality assays, Cox proportional hazards regressions were performed using the PHREG procedure with inhibition category (inhibitors vs. noninhibitors) as the independent variable. Shared variance due to isolate within each inhibition category and across replicate assays was accounted for by using the covs (aggregate) option. The LIFETEST procedure was used to estimate the LT50 of each isolate.

For the nematode infections, chi‐square analyses were used to test whether the probability of mortality and emergence differed between inhibiting and non‐inhibiting isolates. Mixed model analyses of covariance were used to analyze differences in size and number of nematodes between non‐inhibitors and inhibitors. Host mass and either nematode size or number was included as a covariate to test for functional relationships among these traits. Isolate within inhibition status was treated as a random factor. To assess repeatability, chi‐square analyses were performed for each assay, and a Cochran–Mantel–Haenszel test was used to examine the difference between inhibiting and non‐inhibiting isolates while controlling for assay.

## 3 RESULTS

### 3.1 Characterization of symbiont competitive ability

No bacteriocin‐mediated growth inhibition was detected by the bacterial symbionts isolated from four of the nine nematode isolates (Table 1). In contrast, the remaining five isolates were able to inhibit sympatric bacteria associated with conspecific nematodes as well as sympatric bacteria associated with up to three additional *Steinernema* nematode species. No difference was found between these inhibiting and non‐inhibiting isolates in their ability to resist inhibition by other sympatric isolates (19.8 vs. 16.5 out of 28, t = 1.68, *p* = 0.153).

### 3.2 Effect of symbiont status on nematode parasitic success

**Figure 2:**
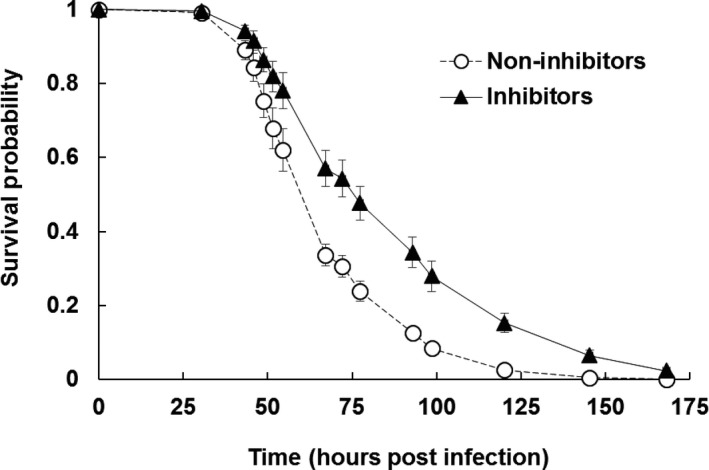
Survival probability of insect hosts (mean ± SE) over hours post infection when infected with non‐inhibiting (white circles, dashed line) and inhibiting (black triangles, solid line) isolates of *Steinernema affine* nematodes. Caterpillars were checked for mortality at given census times. Non‐inhibiting isolates killed caterpillars significantly faster than inhibiting isolates (χ^2^ = 23.68, *df* = 1, *p* < 0.0001).

Nematodes carrying non‐inhibiting isolates killed their insect host faster than those carrying inhibiting isolates (Figure 2, χ^2^ = 23.68, *df *= 1, *p* < 0.0001). This indicates that non‐inhibiting isolates were able to more quickly establish an infection, a key component of reproductive success. There was no difference in probability of host death as nematodes associated with both types of symbionts were highly effective in killing the insect host (non‐inhibitors 95%, inhibitors 88%, χ^2^ = 2.6890, *df *= 1, *p* = 0.1010).

**Figure 3:**
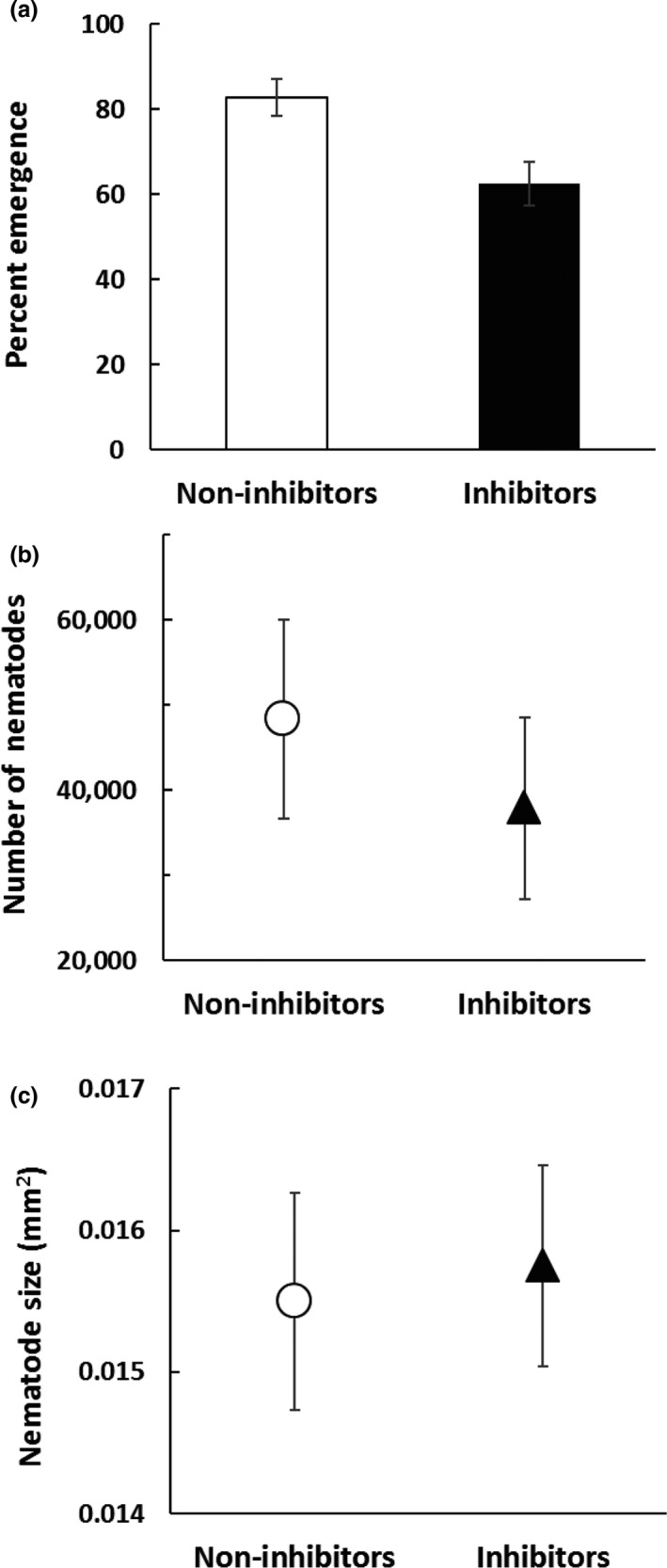
Relative reproductive success of non‐inhibitors and inhibitors measured with (a) percent emergence (calculated as the number of caterpillars that had nematode emergence out of the number of caterpillars that died), χ^2^ = 8.4050, *df* = 1, *p* = 0.0037, (b) number of nematodes that emerged from each caterpillar (*F*_1,7_ = 0.40, *p* = 0.5477), and (c) average nematode size (*F*_1,7_ = 0.06, *p* = 0.8117). Mean ± SE are shown in each panel

Further supporting the hypothesis that the ability to inhibit comes at a cost of reproduction, nematodes carrying non‐inhibiting bacteria were more likely than nematodes carrying inhibiting bacteria to emerge at all once they had successfully killed their insect host (Figure 3a, *df *= 1, χ^2^ = 8.4050, *p* = 0.0037). Additionally, more infective juveniles tended to emerge when the nematodes carried non‐inhibiting bacteria than when they carried inhibiting bacteria (Figure 3b); however, this difference was not significant (*F*_1,7_ = 0.40, *p* = 0.5477). Nor was there a significant difference in the size (area in mm^2^) of emerging nematodes between isolates that carried inhibiting or non‐inhibiting bacteria (Figure 3c, *F*_1,7_ = 0.06, *p* = 0.8178).

### 3.3 Bacterial infection

**Figure 4:**
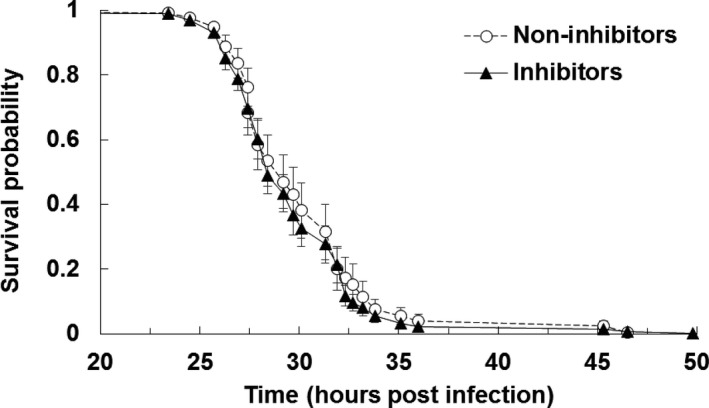
Survival probability of insect hosts (mean ± SE) over hours post infection when infected with non‐inhibiting (white circles, dashed line) and inhibiting (black triangles, solid line) isolates of *Xenorhabdus bovienii* bacteria. Caterpillars were checked for mortality at given census times. There were no significant differences between inhibitors and non‐inhibitors (χ^2^ = 1.4045, *df *= 1, *p* = 0.2360)

When insects were inoculated with the bacteria directly, without their nematodes, the LT50 was found to vary from 26.9 to 32.2 hr across isolates. However, no significant difference was found in the timing of insect host death associate with inhibition phenotypes (Figure 4, *df* = 1, χ^2^ = 1.4045, *p* = 0.2360).

### 3.4 Repeatability of isolate differences in nematode success

**Figure 5:**
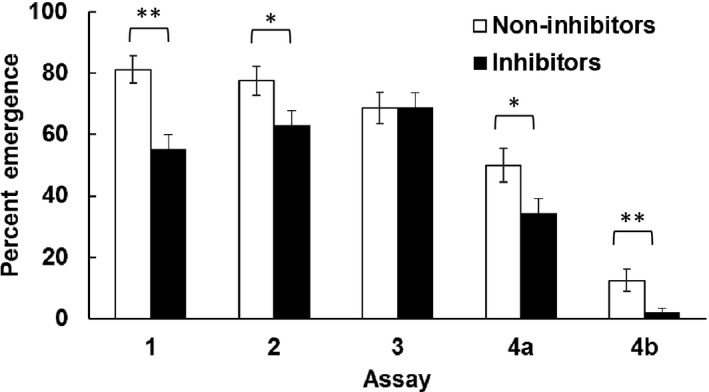
The probability that nematodes carrying non‐inhibiting bacteria (white bars) or inhibiting bacteria (black bars) emerged from caterpillar hosts for each assay described in Table 2. Error bars show the asymptotic standard error. P‐values from chi‐square tests are indicated by *p < 0.05, **p < 0.01.

Based on Assay 1, where non‐inhibitors were faster at killing the insect host and more likely to emerge than inhibitors, we focused on the overall probability of infection success and therefore measured the overall probability of emergence for subsequent assays. Despite differences in nematode age and passaging in the laboratory across Assays 2–4, non‐inhibitors were significantly more likely to successfully infect and reproduce when exposed to an insect than inhibitors (Figure 5, Cochran–Mantel–Haenszel statistic value = 9.8487, *df *= 1, and *p* = 0.0017). Furthermore, this effect was consistent when a second insect host, *M. americanum*, was used in Assay 4b. The only assay which did not show a difference between nematodes carrying inhibiting and non‐inhibiting bacteria effect was Assay 3, where younger IJs were used (1 month old).

## 4 DISCUSSION

Despite profoundly affecting the fitness of their hosts, mutualistic symbionts exhibit a striking amount of variation, even within a single host population (Heath & Stinchcombe, 2014). In this study, we have examined whether variation in the competitive environment can be a mechanism whereby diversity is maintained within a single population. Prior work has demonstrated that bacteriocins produced by *X. bovienii* inhibit the growth of sensitive *Xenorhabdus* competitors (Hawlena, Bashey, & Lively, 2010), and confer a competitive advantage to producing lineages (Bashey et al., 2012) and their nematode hosts (Bashey et al., 2013). Nevertheless, in nature, non‐inhibiting isolates are found with high frequency (e.g., >25% in Hawlena et al, 2012). Here, we categorized the competitive ability of *S. affine* nematode isolates collected along a 300‐m transect and found that 44% were associated with *Xenorhabdus* symbionts that could not inhibit sympatric *Xenorhabdus* competitors (Table 1). We then tested the hypothesis that these non‐inhibiting isolates have a fitness advantage over inhibiting isolates in a non‐competitive context. To do this, we compared infection dynamics of non‐inhibiting and inhibiting isolates via single‐strain infections, both when the nematodes were coupled to their bacterial symbionts and when bacteria were acting alone.

Fitness of entomopathogenic nematodes depends on being able to effectively kill the insect host, reproduce within the carcass, and produce infective juveniles, which emerge and persist in a non‐feeding state until they locate a new insect host (Poinar & Thomas, 1966). Nematodes carrying non‐inhibiting symbionts were quicker to kill (Figure 2) and more likely to have juveniles emerge from their insect hosts (Figures 3 and 5) than nematodes carrying inhibiting symbionts. Thus, there appears to be a trade‐off between an isolate's competitive ability via bacteriocins and its reproductive potential in the absence of competition. We further examined the number and size of infective juveniles emerging from inhibiting and non‐inhibition isolates and found no significant differences in the number or quality of these transmission stage propagules (Figure 3b and c). Together, these results suggest that the difference among these isolates may relate to the probability that the insect will be successfully killed in time to support the reproductive development of the nematodes. Fitness of the bacteria is intimately tied to nematode emergence, as the bacteria rely on the nematodes for vectoring and entry into new insect hosts (Poinar & Thomas, 1966).

This pattern of competitive ability being linked to lower nematode and bacteria fitness suggests that there may be two different strategies for parasite success. Some isolates may adopt a competitive strategy of producing bacteriocin. If a sensitive bacterial strain is present within the insect cadaver, this strategy can confer a competitive advantage for the inhibiting strain (Bashey et al, 2013). However, as seen in our study, if a sensitive strain is not present, inhibitor status may be associated with a reproductive cost. This may either be a direct cost of producing bacteriocin or may be a result of additional evolutionary changes in the genomes linked to this competitive strategy. In contrast to the competitive strategy employed by inhibitors, non‐inhibitors may be employing an alternative strategy focused on establishing an infection quickly, forgoing attack of competitors with bacteriocins in favor of more effective nematode development inside the insect host. Due to the increased reproductive success of non‐inhibitors, we expect non‐inhibitors to dominate in the population except as they are kept in check by being susceptible to a competitor's bacteriocin.

The mechanism underlying the reproductive cost that inhibitors pay is unknown. One potential explanation for the relative decrease in reproductive ability is that inhibiting isolates are producing bacteriocin while non‐inhibiting strains are not. To produce bacteriocin, the producing cell is thought to lyse (Morales Soto et al., 2012), potentially decreasing the overall number of bacterial symbionts available for other symbiont roles. Within insect production and assemblage of the phage‐like bacteriocin particles has been demonstrated in *Xenorhabdus* (Morales‐Soto & Forst, 2011) and may use resources otherwise allocated for killing the insect host or aiding in nematode reproduction. While some bacteria may increase bacteriocin production in response to the presence of competitors, no difference in bacteriocin activity was detected when *Xenorhabdus* was grown with and without a competitor (Bhattacharya, Pak, & Bashey, 2018). Therefore, when in a non‐competitive context, the inhibitors may be paying a cost to produce bacteriocin, but receiving no competitive benefit.

When injected into caterpillars without their nematodes; however, non‐inhibiting and inhibiting bacteria showed no difference in how quickly they could kill the insect host (Figure 4). This result indicates that the costs associated with inhibiting bacteria are dependent on the presence of the nematode. One mechanism whereby this interactive effect could occur is by the bacterial load carried by each nematode (Emelianoff et al., 2008). Non‐inhibiting bacteria may be better able to associate with the nematode than inhibiting bacteria, and therefore changes in infection success may be due to differences in the initial inoculum, or in how the inoculum is retained. In contrast, in the bacteria alone infections, we gave each insect approximately the same dose of bacteria. Thus, differences due to dose would not be seen without the nematodes. Furthermore, nematodes lose some of their bacteria as they age (Flores‐Lara, Renneckar, Forst, Goodrich‐Blair, & Stock, 2007), which could explain why the differences between bacterial types were not seen in the assay done using younger nematodes (Assay 3 in Figure 5), although further testing is needed to support this hypothesis.

Additionally, it is possible that the difference in reproductive success observed between inhibiting and non‐inhibiting isolates arises due to a difference in their symbionts’ ability to support nematode reproductive development. This hypothesis could be addressed by experimentally removing the symbionts and then reassociating the different nematode isolates with their own or different symbionts as has been done elegantly across broader geographic and phylogenetic scales (Chapuis et al., 2009; Murfin et al., 2015; Sicard et al., 2004). Such a study could parse out whether it is just the presence of the nematode or whether specific nematode genotypes are responsible for the observed difference in reproductive success. Coevolution between the nematode and bacterial genomes may limit the ability of a single bacterial genome to evolve competitive and non‐competitive phenotypes simultaneously, and further promote maintenance of diversity in the symbiont population.

Differences between inhibiting and non‐inhibiting isolates could also be due to differences in insect host specialization. While *Steinernema* nematodes are known for their broad host ranges (Peters, 1996; Puza & Mrácek, 2010), it is possible that the bacterial phenotypes are differentially successful on different insect species. We tested this by examining whether patterns observed in the initial insect tested (*Galleria mellonella*) also held in a second host*, Malacosoma americanum*. Both insects showed a similar pattern of lower success of inhibitors, suggesting that insect specialization is not important for the observed symbiont effects. However, as both insects tested are Lepidopteran, it may be important to use hosts from different insect orders to test how inhibitors and non‐inhibitors behave in different host contexts.

Our study demonstrates a symbiont‐mediated trade‐off in host fitness across competitive contexts. Heterogeneity in the competitive environment, thus, may serve to maintain within‐population genetic variation and alter the evolutionary potential of both mutualistic partners. As such, our work highlights the importance of understanding fine‐scale environmental variation and of studying natural isolates in order to gain insights into symbiont phenotypic diversity.

## AUTHOR CONTRIBUTIONS

FB isolated and characterized the organism used in the study; SBM performed the infections and imaging analysis; and SBM and FB designed the study, performed statistical analysis, and wrote the manuscript collaboratively.

## DATA ACCESSIBILITY

Data available from the Dryad Digital Repository: https://doi.org/10.5061/dryad.r5q5v1p

## References

[ece34538-bib-0001] Bashey, F. , Hawlena, H. , & Lively, C. M. (2013). Alternative paths to success in a parasite community: Within‐host competition can favor higher virulence or direct interference. Evolution, 67, 900–907. 10.1111/j.1558-5646.2012.01825.x 23461339

[ece34538-bib-0002] Bashey, F. , Morran, L. T. , & Lively, C. M. (2007). Co‐infection, kin selection, and the rate of host exploitation by a parasitic nematode. Evolutionary Ecology Research, 9, 947–958.

[ece34538-bib-0003] Bashey, F. , Reynolds, C. , Sarin, T. , & Young, S. K. (2011). Virulence and competitive ability in an obligately killing parasite. Oikos, 120, 1539–1545. 10.1111/j.1600-0706.2011.19304.x

[ece34538-bib-0004] Bashey, F. , Young, S. K. , Hawlena, H. , & Lively, C. M. (2012). Spiteful interactions between sympatric natural isolates of *Xenorhabdus bovienii* benefit kin and reduce virulence. Journal of Evolutionary Biology, 25, 431–437. 10.1111/j.1420-9101.2011.02441.x 22221661

[ece34538-bib-0005] Bhattacharya, A. , Pak, H. , & Bashey, F. (2018). Plastic responses to competition: Does bacteriocin production increase in the presence of nonself competitors? Ecology and Evolution, 8, 6880–6888. 10.1002/ece3.4203 30073052 PMC6065276

[ece34538-bib-0006] Boemare, N. E. , Boyer‐Giglio, M. H. , Thaler, J. O. , Akhurst, R. J. , & Brehelin, M. (1992). Lysogeny and bacteriocinogeny in *Xenorhabdus nematophilus* and other *Xenorhabdus* spp. Applied and Environmental Microbiology, 58, 3032–3037. 1444417 10.1128/aem.58.9.3032-3037.1992 PMC183044

[ece34538-bib-0007] Brivio, M. F. , Pagani, M. , & Restelli, S. (2002). Immune suppression of *Galleria mellonella* (Insecta, Lepidoptera) humoral defenses induced by *Steinernema feltiae* (Nematoda, Rhabditida): Involvement of the parasite cuticle. Experimental Parasitology, 101, 149–156. 10.1016/S0014-4894(02)00111-X 12427469

[ece34538-bib-0008] Brock, D. A. , Read, S. , Bozhchenko, A. , Queller, D. C. , & Strassmann, J. E. (2013). Social amoeba farmers carry defensive symbionts to protect and privatize their crops. Nature Communications, 4 10.1038/ncomms3385 24029835

[ece34538-bib-0009] Chapuis, É. , Emelianoff, V. , Paulmier, V. , Le Brun, N. , Pagès, S. , Sicard, M. , & Ferdy, J. B. (2009). Manifold aspects of specificity in a nematode–bacterium mutualism. Journal of Evolutionary Biology, 22, 2104–2117. 10.1111/j.1420-9101.2009.01829.x 19732258

[ece34538-bib-0010] Clay, K. , & Fox, C. (2014). Defensive symbiosis: A microbial perspective. Functional Ecology, 28, 293–298. 10.1111/1365-2435.12258

[ece34538-bib-0011] Emelianoff, V. , Chapuis, E. , Le Brun, N. , Chiral, M. , Moulia, C. , & Ferdy, J.‐B. (2008). A survival‐reproduction trade‐off in entomopathogenic nematodes mediated by their bacterial symbionts. Evolution, 62, 932–942. 10.1111/j.1558-5646.2008.00319.x 18194474

[ece34538-bib-0012] Feldhaar, H. (2011). Bacterial symbionts as mediators of ecologically important traits of insect hosts. Ecological Entomology, 36, 533–543. 10.1111/j.1365-2311.2011.01318.x

[ece34538-bib-0013] Flores‐Lara, Y. , Renneckar, D. , Forst, S. , Goodrich‐Blair, H. , & Stock, P. (2007). Influence of Nematode Age and Culture Conditions on Morphological and Physiological Parameters in the Bacterial Vesicle of Steinernema carpocapsae (Nematoda : Steinernematidae). Journal of Invertebrate Pathology, 95, 110–118. 17376477 10.1016/j.jip.2007.01.006

[ece34538-bib-0014] Forst, S. , & Nealson, K. (1996). Molecular biology of the symbiotic pathogenic bacteria *Xenorhabdus* spp. and *Photorhabdus* spp. Microbiological Reviews, 60, 21–43. 8852894 10.1128/mr.60.1.21-43.1996 PMC239416

[ece34538-bib-0015] Götz, P. , Boman, A. , & Boman, H. G. (1981). Interactions between insect immunity and an insect‐pathogenic nematode with symbiotic bacteria. Proceedings of the Royal Society B: Biological Sciences, 212(1188), 333–350. 10.1098/rspb.1981.0043

[ece34538-bib-0016] Hawlena, H. , Bashey, F. , & Lively, C. M. (2010). The evolution of spite: Population structure and bacteriocin‐mediated antagonism in two natural populations of *Xenorhabdus* bacteria. Evolution, 64, 3198–3204. 10.1111/j.1558-5646.2010.01070.x 20584073

[ece34538-bib-0017] Hawlena, H. , Bashey, F. , & Lively, C. M. (2012). Bacteriocin‐mediated interactions within and between coexisting species. Ecology and Evolution, 2, 2521–2526. 10.1002/ece3.354 23145336 PMC3492777

[ece34538-bib-0018] Hawlena, H. , Bashey, F. , Mendes Soares, H. , & Lively, C. M. (2010). Spiteful interactions in a natural population of the bacterium *Xenorhabdus bovienii* . The American Naturalist, 175, 374–381. 10.1086/650375 20095826

[ece34538-bib-0019] Heath, K. D. (2010). Intergenomic epistasis and coevolutionary constraint in plants and rhizobia. Evolution, 64, 1446–1458. 10.1111/j.1558-5646.2009.00913.x 20002161

[ece34538-bib-0020] Heath, K. D. , & Stinchcombe, J. R. (2014). Explaining mutualism variation: A new evolutionary paradox? Evolution, 68, 309–317. 10.1111/evo.12292 24303853

[ece34538-bib-0021] Laforsch, C. , & Tollrian, R. (2004). Inducible defenses in multipredator environments: Cyclomorphosis in *Daphnia cucullata* . Ecology, 85, 2302–2311. 10.1890/03-0286

[ece34538-bib-0022] Lankau & Strauss, S., (2007). Mutual feedbacks maintain both genetic and species diversity in a plant community. Science, 317, 1561–1563. 10.1126/science.1147455 17872447

[ece34538-bib-0023] Lankau, R. (2008). A chemical trait creates a genetic trade‐off between intra‐ and interspecific competitive ability. Ecology, 89, 1181–1187. 10.1890/07-1541.1 18543611

[ece34538-bib-0024] Lively, C. M. (1986). Predator‐induced shell dimorphism in the acorn barnacle *Chthamalus ansiopma* . Evolution, 40, 232–242. 28556032 10.1111/j.1558-5646.1986.tb00466.x

[ece34538-bib-0025] Martens, E. C. , Heungens, K. , & Goodrich‐Blair, H. (2003). Early colonization events in the mutualistic association between *Steinernema carpocapsae* nematodes and *Xenorhabdus nematophila* bacteria. Journal of Bacteriology, 185, 3147–3154. 10.1128/JB.185.10.3147-3154.2003 12730175 PMC154081

[ece34538-bib-0026] Morales‐Soto, N. , & Forst, S. A. (2011). The xnp1 P2‐like tail synthesis gene cluster encodes xenorhabdicin and is required for interspecies competition. Journal of Bacteriology, 193, 3624–3632. 10.1128/JB.00092-11 21602326 PMC3133312

[ece34538-bib-0027] Morales‐Soto, N. , Gaudriault, S. , Ogier, J. C. , Thappeta, K. R. V. , & Forst, S. (2012). Comparative analysis of P2‐type remnant prophage loci in Xenorhabdus bovienii and Xenorhabdus nematophila required for xenorhabdicin production. FEMS Microbiology Letters, 333, 69–76. 10.1111/j.1574-6968.2012.02600.x 22612724

[ece34538-bib-0028] Morgan, J. A. W. , Kuntzelmann, V. , Tavernor, S. , Ousley, M. A. , & Winstanley, C. (1997). Survival of *Xenorhabdus nematophilus* and *Photorhabdus luminescens* in water and soil. Journal of Applied Microbiology, 83, 665–670. 10.1046/j.1365-2672.1997.00281.x

[ece34538-bib-0029] Murfin, K. E. , Lee, M. M. , Klassen, J. L. , McDonald, B. R. , Larget, B. , Forst, S. , … Goodrich‐Blair, H. (2015). *Xenorhabdus bovienii* Strain Diversity Impacts Coevolution and Symbiotic Maintenance with Steinernema spp. Nematode Hosts. Mbio, 6, e00076–e115. 26045536 10.1128/mBio.00076-15 PMC4462624

[ece34538-bib-0030] Oliver, K. M. , Campos, J. , Moran, N. A. , & Hunter, M. S. (2008). Population dynamics of defensive symbionts in aphids. Proceedings of the Royal Society B: Biological Sciences, 275, 293–299. 10.1098/rspb.2007.1192 18029301 PMC2593717

[ece34538-bib-0031] Peters, A. (1996). The natural host range of *Steinernema* and *Heterorhabditis* spp. and their impact on insect populations. Biocontrol Science and Technology, 6, 389–402. 10.1080/09583159631361

[ece34538-bib-0032] Poinar, G. O. J. , & Thomas, G. M. (1966). Significance of *Achromobacter nematophilus* Poinar and Thomas (Achromobacteraceae: Eubacteriales) in the development of the nematode, DD‐136 (*Neoplectana* sp. Steinernematidae). Parasitology, 56, 385–390. 4960247 10.1017/s0031182000070980

[ece34538-bib-0033] Popiel, I. , Grove, D. L. , & Friedman, M. J. (1989). Infective juvenile formation in the insect parasitic nematode *Steinernema feltiae* . Parasitology, 99, 77–81. 10.1017/S0031182000061047

[ece34538-bib-0034] Puza, V. , & Mrácek, Z. (2010). Mechanisms of coexistence of two sympatric entomopathogenic nematodes, *Steinernema affine* and *S. kraussei* (Nematoda: Steinernematidae), in a central European oak woodland soil. Applied Soil Ecology, 45, 65–70. 10.1016/j.apsoil.2010.02.002

[ece34538-bib-0035] Qui, L. , & Bedding, R. (2000). Energy metabolism and its relation to survival and infectivity of infective juveniles of *Steinernema carpocapsae* under aerobic conditions. Nematology, 2, 551–559. 10.1163/156854100509330

[ece34538-bib-0036] Riley, M. A. , & Chavan, M. A. (2007). Bacteriocins: Ecology and Evolution (p. 150). Berlin: Springer‐Verlag.

[ece34538-bib-0037] Sicard, M. , Ferdy, J. B. , Pages, S. , Le Brun, N. , Godelle, B. , Boemare, N. , & Moulia, C. (2004). When mutualists are pathogens: An experimental study of the symbioses between Steinernema (entomopathogenic nematodes) and *Xenorhabdus* (bacteria). Journal of Evolutionary Biology, 17, 985–993. 10.1111/j.1420-9101.2004.00748.x 15312071

[ece34538-bib-0038] Sicard, M. , Le Brun, N. , Pages, S. , Godelle, B. , Boemare, N. , & Moulia, C. (2003). Effect of native *Xenorhabdus* on the fitness of their *Steinernema* hosts: Contrasting types of interactions. Parasitology Research, 91, 520–524. 10.1007/s00436-003-0998-z 14557877

[ece34538-bib-0039] Simões, N. (2000). Pathogenicity caused by high virulent and low virulent strains of *Steinernema carpocapsae* to *Galleria mellonella* . Journal of Invertebrate Pathology, 75, 47–54. 10.1006/jipa.1999.4899 10631057

[ece34538-bib-0040] Snyder, H. , Stock, S. P. , Kim, S. , Flores‐Lara, Y. , & Forst, S. (2007). New insights into the colonization and release process of *Xenorhabdus nematophila* and the morphology and ultrastructure of the bacterial receptacle of its nematode host *Steinernema carpocapsae* . Applied and Environmental Microbiology, 73, 5338–5346. 17526783 10.1128/AEM.02947-06 PMC1951000

[ece34538-bib-0041] Stock, S. P. , Campbell, J. F. , & Nadler, S. (2001). Phylogeny of *Steinernema* Travassos, 1927 (Cephalobina: Steinernematidae) inferred from ribosomal DNA sequences and morphological characteristics. Journal of Parasitology, 87, 877–889. 11534654 10.1645/0022-3395(2001)087[0877:POSTCS]2.0.CO;2

[ece34538-bib-0042] Strauss, S. Y. , Rudgers, J. A. , Lau, J. A. , & Irwin, R. E. (2002). Direct and ecological costs of resistance to herbivory. Trends in Ecology & Evolution, 17, 278–285. 10.1016/S0169-5347(02)02483-7

[ece34538-bib-0043] Tailliez, P. , Pages, S. , Ginibre, N. , & Boemare, N. (2006). New insight into diversity in the genus *Xenorhabdus*, including the description of ten novel species. International Journal of Systematic and Evolutionary Microbiology, 56, 2805–2818. 10.1099/ijs.0.64287-0 17158981

[ece34538-bib-0044] Therese, M. O. , & Bashey, F. (2012). Natal‐host environmental effects on juvenile size, transmission success, and operational sex ratio in the entomopathogenic nematode *Steinernema carpocapsae* . Journal of Parasitology, 98, 1095–1100. 22663291 10.1645/GE-3069.1

[ece34538-bib-0045] Wang, J. , & Bedding, R. (1996). Population development of *Heterorhbditis bacteriophora* and *Steinernema carpocapsae* in the larvae of *Galleria mellonella* . Fundamental Applications of Nematology, 19, 363–367.

[ece34538-bib-0046] White, G. F. (1927). A method for obtaining nematode larvae from cultures. Science, 66, 302–303. 10.1126/science.66.1709.302-a 17749713

